# 3D-Printable Biopolymers for Socket Preservation Technique: Soft Tissues Response: A Pilot Randomised Clinical Study

**DOI:** 10.3390/dj12100321

**Published:** 2024-10-07

**Authors:** Nicola De Angelis, Paolo Pesce, Wiwiek Poedjiastoeti, Trijani Suwandi, Rosalina Tjandrawinata, Francesco Bagnasco, Maria Menini

**Affiliations:** 1 Unit of Prosthodontics and Implant Prosthodontics, Department of Surgical Sciences and Integrated Diagnostics, University of Genoa, 16126 Genova, Italy; paolo.pesce@unige.it (P.P.); fcbagna5@hotmail.it (F.B.); maria.menini@unige.it (M.M.); 2 Department of Periodontology, Faculty of Dentistry, Universitas Trisakti, Jakarta 11440, Indonesia; trijani@trisakti.ac.id; 3 Department of Oral and Maxillofacial Surgery, Dean Faculty of Dentistry, Universitas Trisakti, Jakarta 11440, Indonesia; wiwiek@trisakti.ac.id; 4 Department of Dental Materials, Universitas Trisakti, Jakarta 11440, Indonesia; rosalina@trisakti.ac.id

**Keywords:** extraction socket, socket preservation, 3D printing, biopolymers

## Abstract

Background: The aim of the present parallel clinical study is to evaluate the efficacy of 3D-printed biopolymers compounded with osteoconductive material (beta-tricalcium phosphate and hydroxyapatite) for soft tissue closure after tooth extraction. Materials and Methods: this study followed the CONSORT reporting guidelines; 39 patients were treated with socket preservation using 3D-printed biopolymers and randomly divided into 3 groups (Test 1, Test 2, and Control). All cases were treated without flap elevation, careful cleaning and debridement of the sites, and then randomly sealed as follows: In T1, with a 3D-printed disk of poli-D-lactic acid with 10% of hydroxyapatite; in T2, using a 3D-printed disk of poli-ε caprolactone with 20% of β-tricalcium phosphate; and in T3, the socket was left open to heal. At baseline (extraction time) and 6 weeks after extraction, the rate of exposure was evaluated and stratified according to the site (anterior, posterior). Results: No dropouts were observed during the 6 weeks follow-up. All sites underwent uneventful healing with no complications. For posterior teeth, Test 1 and Test 2 showed full healing of the soft tissues with a reduction of the exposed area from 46.5 ± 8.25 mm^2^ to 0.6 ± 0.84 mm^2^ and from 47.1 ± 8.67 mm^2^ to 0.6 ± 0.7 mm^2^, respectively. The Control group exhibited a reduction from 45.6 ± 7.25 mm^2^ to 1.2 ± 0.9 mm^2^. Both Tests 1 and 2, when compared to the Control group, showed statistically significant better healing (*p* < 0.05). Anterior teeth showed a complete closure of the socket 4 weeks after the extraction with no noticeable differences between Test and Control. Conclusions: Both materials used in this study showed evidence to achieve the purpose. Ethical Guidelines: written informed consent was obtained from the participants of the study, as requested by the Ethics Committee for Health Research Faculty of Dentistry, Universitas Trisakti, with the following number: 641/S3/KEPK/FKG/5/2023.

## 1. Introduction

By 2050, it is estimated that 8.6 million individuals in the United States will experience edentulism [1], with even more severe impacts anticipated in developing nations. According to WHO guidelines [2], individuals aged 35 to 45 face the highest prevalence of partial edentulism, often as a result of inadequate access to dental care. Without timely intervention, this condition frequently progresses to complete edentulism in older populations. The underlying causes of tooth loss, which include trauma, periodontal disease, and oral cancers, can lead to significant bone deficiencies, exacerbating the challenges of rehabilitation and quality of life for affected individuals.

A bone defect is defined as an anatomical condition which does not allow the conventional placement of implants [1]. In order to restore the lost anatomy and function, alveolar bone augmentation is often required. While significant progress has been made in recent decades, several challenges remain regarding hard-tissue augmentation procedures.

Scaffolds include both non-resorbable and resorbable membranes as well as titanium meshes. Non-resorbable membranes, considered the first generation of barriers, have been extensively studied and clinically tested for several years. They are primarily made from polytetrafluoroethylene and titanium grade 5 alloy (Ti-6Al-4V), materials known for their excellent biocompatibility and structural integrity throughout the implantation period [3].

Two main drawbacks must be considered: the need for removal via a second surgical procedure after adequate bone volume has been restored, which poses a risk to the newly regenerated bone tissue, and the potential exposure of the membrane to the oral environment, which increases the risk of secondary infections [2,3].

In contrast, resorbable membranes have been developed primarily to eliminate the need for a second surgery. Furthermore, in the event of exposure during the healing phase, these membranes can be rapidly resorbed, mitigating the risk of infection [4]. The materials used in their production may be natural (such as collagen and chitosan), which offer excellent biocompatibility and enhance wound healing and bone formation, or synthetic, mainly poly(L-lactide) or poly(L-lactide-co-glycolide), which are the most widely studied and clinically used bioresorbable polymers approved by the FDA.

Socket preservation, on the other hand, is a technique that intentionally leaves resorbable membranes exposed during the healing phase [5]. The type of graft, as well as the membrane used in the procedure, may yield different outcomes, as highlighted in previous systematic reviews [6,7].

While autologous bone grafts are typically considered the gold standard, they come with disadvantages, including the need for a donor site, increased surgical time, and higher morbidity. As a result, alternative materials such as allografts or xenografts are often preferred [8]. Currently, xenograft materials stand out as one of the premier options for alveolar preservation following tooth extraction. Their superior clinical results and ease of use make them highly favoured among practitioners.

The sealing technique also offers a variety of materials, ranging from autogenous-free gingival grafts to collagen matrices or synthetic membranes [9]. Titanium meshes are the only metal-based devices used as barrier membranes for guided bone regeneration [6]. Due to their rigidity, titanium meshes need to be shaped and adapted to the bone defect during surgery. This sensitive, time-consuming, and laborious step increases the duration of surgery, with the final outcome largely dependent on the operator’s skill.

In recent years, titanium alloys have also been employed in additive manufacturing processes to create customised devices for bone regeneration [10]. Meshes obtained through 3D printing offer several surgical advantages, including precision, rigidity, and rounded corners and margins. However, they also present some limitations, such as the lack of direct interaction with the mesh design by the operator, the necessity of removal after several months, unclear effects of post-production treatment on the mesh surface [11,12,13], and high costs (averaging 200 € per mesh).

Among the different 3D printing techniques, fused deposition modelling (FDM), which involves the extrusion of a filament into layers to create a three-dimensional object, offers the potential to design and fabricate, at relatively low cost, highly reproducible, bioresorbable 3D scaffolds with a fully interconnected pore network. Typical materials used in FDM with biodegradable and bioresorbable properties include poly(lactic acid) (PLA) and poly(ε-caprolactone) (PCL) [14].

The scope of the present research, which to the authors’ knowledge is the first study to explore the use of 3D-printed biopolymers for sealing extraction sockets, was to evaluate, with short-term follow-up, the clinical outcomes of a socket preservation technique using 3D-printable biopolymers. The aim was to present a novel approach for bone augmentation procedures based on the printing of fully resorbable polymeric devices, thereby avoiding the need for a second surgery and offering a more affordable solution. Specifically, the null hypothesis tested was that in socket preservation procedures, no differences would be observed in soft-tissue healing when using two different 3D-printed biopolymers or no grafting materials.

## 2. Materials and Methods

**Figure 1:**
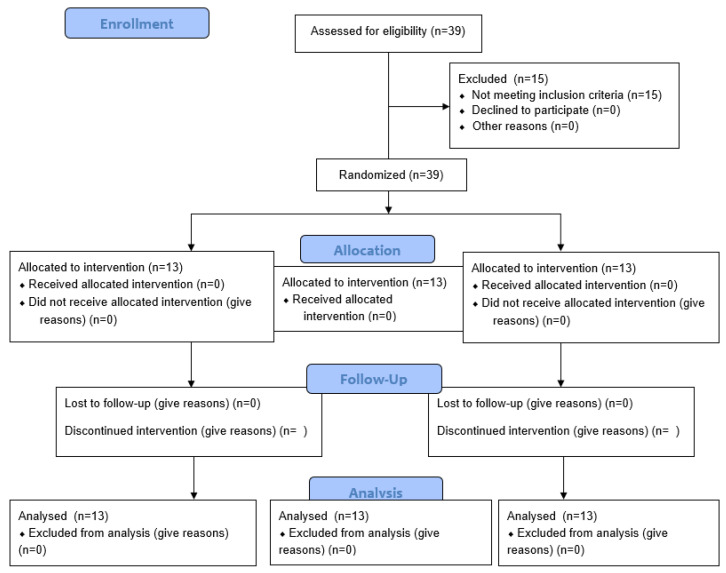
CONSORT 2010 Flow Diagram.

This parallel clinical study followed the CONSORT reporting guidelines [15] and was conducted in the University Department in Trisakti, Indonesia. The protocol has been evaluated and cleared by the Ethics Committee for Health Research Faculty of Dentistry, Universitas Trisakti, with the following number: Clin. Tr. 641/S3/KEPK/FKG/5/2023. This clinical trial has been registered at the ISRCTN registry with registration number ISRCTN12199305 on 4 March 2024. All the proposed treatments were conducted according to the Declaration of Helsinki for human rights, and CONSORT Guidelines have been followed (Figure 1). 

### 2.1. Sample Size Calculation

For the calculation of the sample size, the following formula was used:

$$n=\left[{\left(\frac{Z\alpha }{2}+Z\beta \right)}^{2}\times \frac{\sigma^{2}+\sigma^{2}+\sigma^{2}}{{\left(\Delta \right)}^{2}}\right]+\left(m-1\right)\times k$$

where:

*n* is the total size for all groups,
*Zα*/2 is the critical value for the level of significance (5%) = 1.96,
*Zβ* is the critical value related to the power (80%) = 0.84,
*σ* is the standard deviation in every group = 0.5,
Δ is the clinically minimal difference between the groups = 0,
*m* is the number of planned treatments = 3,
*k* is the correction of dropouts = 1.

A total of 39 patients were requested to start the study, and all the data of the equation above were retrieved in the context of clinical trials and epidemiological studies on ClinCalc and other medical research platforms, although no one is referring to the material used for this specific investigation.

### 2.2. Patients Enrolment

Patients with no exclusion of sex or race, >18 years old, and who had at least one tooth to be extracted, were consecutively enrolled from 1 June 2023 to 31 July 2023. The subjects had to be medically healthy, with no assumption of bifosphonates and no or light–medium smokers (maximum 10 cigarettes/day). Pregnancy and lactation were considered as exclusion criteria.

Patients with signs of acute infection at the extraction site were not included, while chronic apical infectious sites were similarly enrolled in the study.

After the enrolment all the subjects were randomly allocated to 3 different groups by using the software www.randomizer.org (1997–2024 by Geoffrey C. Urbaniak and Scott Plous, accessed on 9 July 2024).

One week before extraction all the patients underwent a professional oral hygiene session.

**Figure 2:**
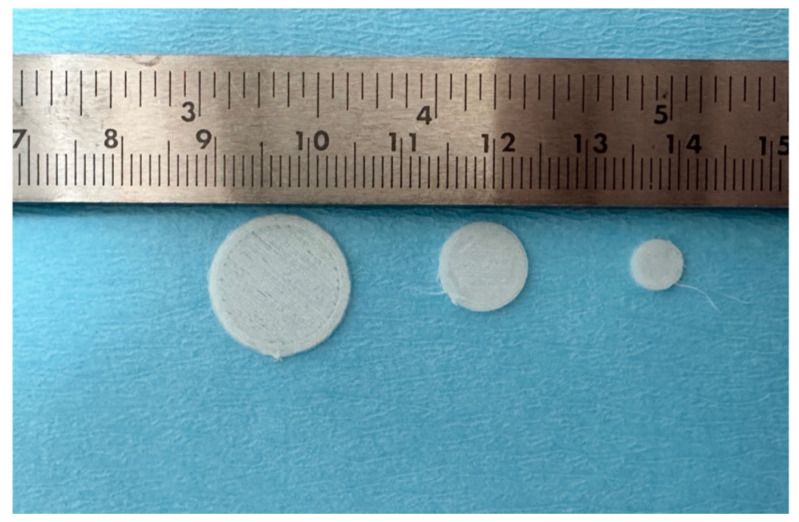
Polymeric compounded disks 3D printed with 3 different diameters: 4, 6, and 12 mm.

Two different bioco-polymers have been employed, derived from the most common bio-polymers: poli-D-lactic acid with the addition of 10% hydroxyapatite and poli-ε caprolactone with 20% β-tricalcium phosphate for the closure of the extraction sockets [16]. These materials were 3D printed (FDM technique after CAD design) in disks with different diameters and the same thickness of 0.8 mm. (4 and 6 mm for anterior teeth and 12 mm for posterior sites), so that the appropriate size could be chosen based on the dimension of the extraction sockets (Figure 2). 

### 2.3. Surgical Treatment

All the extractions were performed without flap elevation and without any antibiotic prophylaxis.

A careful cleaning of the socket was conducted in order to exclude the possible presence of inflammatory tissue without grafting any additional bone substitute. Once tooth extraction was completed, the operator opened a sealed envelope containing the result of the randomisation procedure in order to include the patient in one of the following study groups (Figure 1):

- TEST 1: a 3D-printed disk of poli-D-lactic acid with 10% of hydroxyapatite had to be trimmed inside the gingival margin and ensured with a crossed mattress suture.
- TEST 2: a 3D-printed disk of poli-ε caprolactone with 20% of β-tricalcium phosphate had to be trimmed inside the gingival margin and ensured with a crossed mattress suture.
- CONTROL: extraction left to heal without any graft materials. Only a collagen sponge was used in case of excessive bleeding [17].

The patients were prescribed to follow a soft and cold diet the day of surgery and pain killers (NAS) were prescribed to be used if necessary.

Patients were instructed to avoid rinsing during the first day after extraction and then in the following days to use Chlorexdine 0.20% three times a day for 7 days.

### 2.4. Follow-Up

Follow-up schedule was as following:

- T0: extraction and socket preservation in test 1 and test 2 groups
- T1 (10 days after extraction): suture removal
- T2 (45 days after extraction): check and picture

### 2.5. Data Extraction and Evaluation

Occlusal pictures of the post-extractive site were taken at every step of the research (T0, T1, and T2) in order to evaluate the progression of soft tissue healing. Pictures were taken with Nikon Reflex D7500 (Tokyo, Japan) equipped with a 105 lens and R1c1 lights.

**Figure 3:**
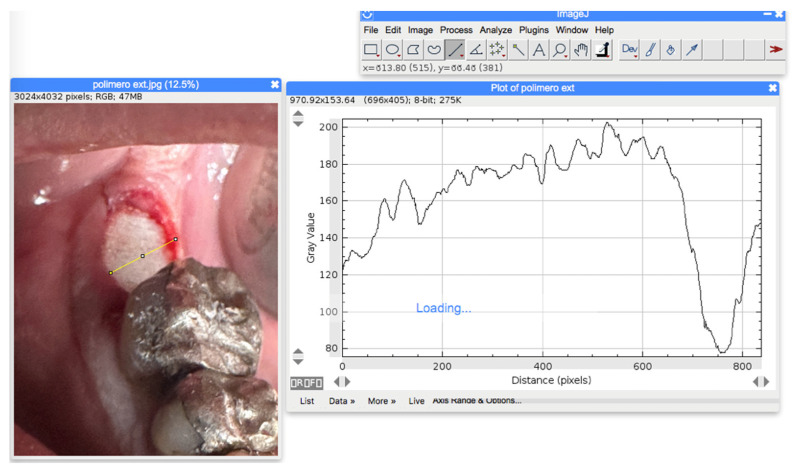
Calculation of the area of the sockets with Image J.

A blind examiner received all the clinical photos and with the use of the open-source software Image J (https://imagej.net, accessed on 9 July 2024),the distance between the buccal and the lingual gum margin and from mesial to distal margin at the post-extractive site were measured in pixels and then converted in millimetres to calculate the area, following the equalisation generated by means of the same software (Figure 3).

Progressive soft-tissue closure as well as the presence/absence of inflammatory signs such as pain and swelling and other possible complications (such alveolitis, suppuration, etc.) were considered as primary outcome and both analysed and stratified according to the different study groups.

### 2.6. Statistical Analysis

A *t*-test was performed by using the software JMP v17.2. Settings included the null hypothesis H0 (no differences between the 3 groups) and H1 (at least one group has a mean different from the others). All data were recorded in a measurable way and the software calculated the mean, the SD and eventually the *t* value. *p* was set at 0.05 significance level.

## 3. Results

This study started in June 2023 and was completed at the end of July 2023.

Thirty-nine patients (20 males and 19 females, aged 20–70 years), 13 per group, were enrolled in the study, and there were no dropouts.

No complications occurred (infection, swelling, pain) throughout the duration of the follow-up (6 weeks), and all the patients anecdotally reported to be satisfied with the treatment received. The total number of extracted teeth was 39: 10 molars (4 upper and 6 lower), 20 premolars (10 upper and 10 lower), and 9 front teeth (5 upper and 4 lower incisors). The mean of the initial exposed area was 46.5 ± 8.25 mm^2^; 47.1 ± 8.67 mm^2^, and 45.6 ± 7.25 mm^2^ on Group 1, Group 2, and the Control group, respectively. At the end of the observation (6 weeks), the mean exposed area was 0.6 ± 0.84 mm^2^, 0.6 ± 0.7 mm^2^, and 1.2 ± 0.9 mm^2^ for Group 1, 2, and the Control group, respectively (Table 1).

Student *t*-tests were performed for the stratification of the results for anterior and posterior teeth and the polymer type with the primary outcome.

The first analysis compared the outcomes between the three groups. The *t* value was 0.5 (*p* > 0.05) when comparing Groups 1 and 2 and −5.4 (*p* < 0.05) and −6.6 (*p* < 0.05) when Groups 1 and 2 were compared with the Control group, respectively.

The second stratification was conducted for posterior sites between the three groups. For the comparison between Groups 1 and 2, the *t* value was 1.16 (*p* > 0.05); comparing Group 1 with Control, the t value was −3.4 (*p* < 0.05); for the comparison between Group 2 and Control, the *t* value was −2.6 (*p* < 0.05).

The third stratification was conducted for anterior sites where the comparison between the Test Groups and Control ones had a *t* value of 1.1 (*p* > 0.05).

**Figure 4:**
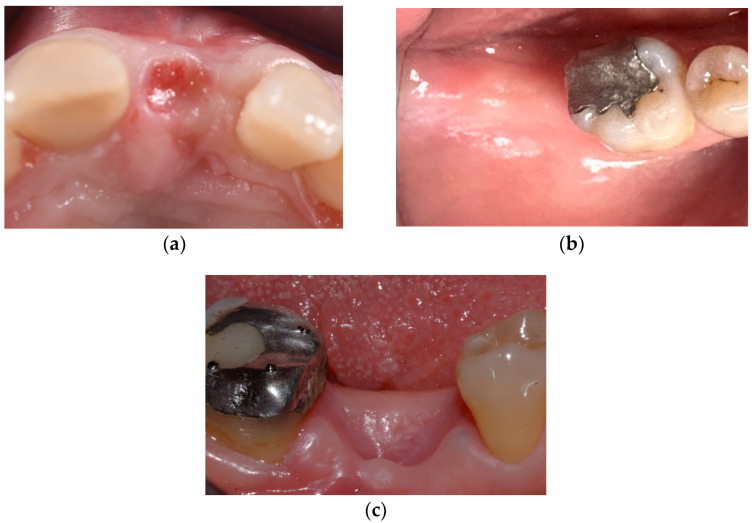
4 weeks observation. (**a**) Test 1; (**b**) Test 2; (**c**) Control.

The observation period was 4 weeks, and the total resorption of the disks and almost a complete closure of the sockets was observed in all the patients included, with more remarkable evidence in Group 1 and Group 2 (Figure 4).

Anterior teeth showed a complete closure of the socket 4 weeks after the extraction with no noticeable differences between Test and Control.

**Figure 5:**
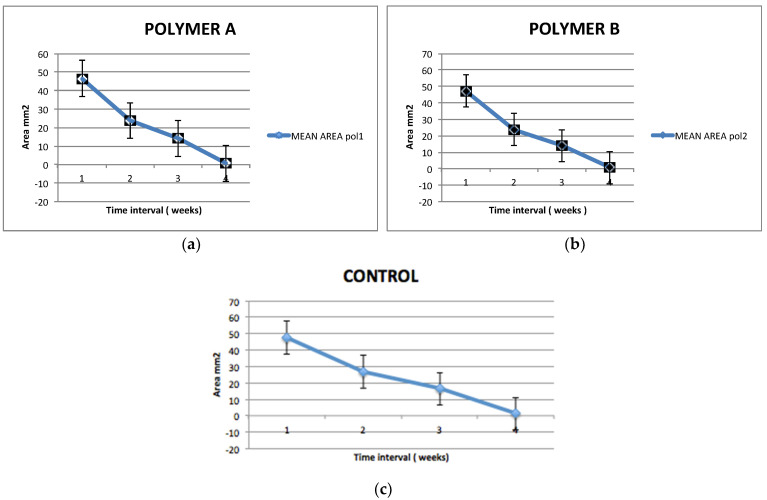
Distance between the buccal and lingual gingival margin at the post-extractive sites for the 3 study groups during the first 4 weeks of healing. (**a**) Polymer A is poli-D-lactic acid with 10% of hydroxyapatite; (**b**) polymer B is poli-ε caprolactone with 20% of β-tricalcium phosphate; (**c**) is the Control group.

The graphs in Figure 5 describe the reduction of the area of exposure of the biopolymer disk throughout the first 4 weeks., with the full resorption of the polymeric disk.

## 4. Discussion

In recent years, polylactic acid (PLA) has emerged as one of the leading FDA-approved biomaterials in the biomedical field, owing to its favorable properties, such as being a thermoplastic and bioresorbable polymer with good mechanical behavior. From a biological perspective, the resorption pattern is critical, as for optimal bone growth, a device must remain intact for at least 4 to 6 months [18]. PLA’s Young’s modulus is approximately 0.3, with a tensile strength ranging from 50 to 70 MPa. Due to its low elongation at break and a glass transition temperature (Tg) close to 60 °C, PLA is considered a brittle material, limiting its use in applications requiring high plastic deformations under elevated stress levels [19]. As a result, PLA can be combined with other materials to reduce its brittleness, such as hydroxyapatite (HA), tricalcium phosphate (TCP), brushite, and monetite [20].

Hydroxyapatite, the primary component and crystal structure of human hard tissues, can be utilized as an additive to modify commonly 3D-printed biological raw materials, enhancing their mechanical properties and osteogenic activity. However, the resorption rate of HA is significantly slower compared to TCP, which is well known for its synergy with surrounding tissues and its ability to induce osteoconductivity. Due to its excellent mechanical properties and remarkable bone remodeling capacity, β-TCP is highly useful in combination with PLA polymer for creating tissue scaffolds.

Dicalcium phosphate dihydrate (brushite) cement is another biocompatible material that can be resorbed under physiological conditions. In vivo studies investigating the biological reaction to, and degradation of, brushite cements have reported either complete or extensive resorption alongside fragmentation or long-term stability of the cement [21]. Crystallographic and spectroscopic analyses of retrieved brushite cement implants, however, have revealed a marked reduction in the resorption rate following the formation of hydroxyapatite within the cement, thought to result from the hydrolysis of brushite [22].

While dicalcium phosphate anhydrous (monetite) shares a similar chemical composition with brushite, its behavior in vivo differs substantially, primarily due to differences in water solubility at physiological pH. Monetite does not reprecipitate into HA in vivo, and recent animal studies have demonstrated its strong osteoconductive and osteoinductive properties, as well as its significant resorption in vivo [23].

Polycaprolactone (PCL), another aliphatic polymer approved by the FDA, belongs to the polyester family and is one of the most widely used polymers in the biomedical field. Unlike PLA, PCL has low tensile strength (about 23 MPa) but exhibits high elongation at break (4700%), granting it a highly elastic behavior and a Young’s modulus of 0.2 to 0.4 GPa, which is significantly lower than PLA’s [24]. PCL has demonstrated excellent biocompatibility, albeit slightly lower than polylactides. Nonetheless, PCL is still widely employed in the biomedical field due to its higher stability [25]. The polymer degrades fully over 2–3 years, a relatively long period, though this can be shortened by combining PCL with other materials such as HA or tricalcium phosphates [26].

The key point in preserving socket volume, and thus facilitating subsequent implant treatment, is to maintain as much pre-existing hard and soft tissue as possible. A critical factor in successful implant-supported rehabilitation is the presence of sufficient bone volume around the implant [27,28]. This is why socket preservation techniques are becoming increasingly popular, and new high-performance materials may help clinicians achieve effective fixed rehabilitations in edentulous areas. A recent study [18] reviewed several systematic analyses with the consensus being that grafts and simple blood clots perform better when a sealing system (resorbable membrane) is placed over the extraction site.

Another recent clinical study [19] presented results of a sealing system infused with growth factors, such as PDGF, directly applied to the membranes. The outcomes were similar to using barriers alone. However, one common challenge with using grafts and resorbable or non-resorbable membranes is the additional cost. Thus, personalized medicine efforts should focus on employing cost-effective, widely available materials [1].

Biopolymers and their co-polymers, with or without ceramic components, are widely used in various medical fields [20]. Today, combining these materials with 3D printing technology offers a viable solution to achieve precision, biocompatibility, and cost efficiency, as highlighted in a recently published paper [21]. The authors compared the in vitro differentiation and maturation of a human pre-osteoblast line on 3D-printed disks of polycaprolactone with 20% β-tricalcium phosphate versus polycaprolactone alone. The findings demonstrated that the composite material showed excellent potential for osteoconduction and did not hinder cell differentiation and maturation.

This study aims to introduce composite co-polymers into clinical practice by investigating tissue response. As detailed in the materials section, two different bio-copolymers were utilized, derived from common biopolymers poly-D-lactic acid with 10% hydroxyapatite and polycaprolactone with 20% β-tricalcium phosphate for closing extraction sockets. These materials were 3D-printed into disks of various diameters to match the size of the extraction sockets.

The first outcome examined was the efficacy of adding a sealing system compared to allowing sockets to heal without coverage. Results showed a progressive reduction in the exposed area, and after six weeks, both polymers achieved nearly complete soft-tissue closure in almost all clinical cases. Meanwhile, in the Control group, tissue healing followed an uncontrolled pattern, leading to irregular margins and notable crestal depressions. Consequently, the null hypothesis cannot be accepted. This result was anticipated, as it aligns with previous studies on site preservation techniques [22,23,24,25]. Both materials displayed similar behavior during the observation period, and by the fourth week, they were no longer detectable inside the socket.

The second outcome focused on the efficacy of coverage in posterior sites (molars and premolars). Results indicated a significant benefit from adding sealing, compared to the Control sites. The two polymeric materials behaved similarly, with no statistically significant difference between Test 1 and Test 2.

In contrast, for anterior teeth, no differences were found between the three groups, suggesting that sealing might not be necessary after extraction. Here, the null hypothesis is accepted. One possible explanation is the smaller diameter of the socket, which might heal spontaneously without additional treatment.

Future research should explore the total volume of the site, as other preservation techniques have [26,27], though this was outside the scope of this study.

A key finding of this investigation was the total absence of post-operative complications, such as inflammatory reactions or infections. Although no literature exists on the tested materials’ behavior, two recent studies [28,29] assessed cellular responses over guided bone regeneration (GBR) barriers, demonstrating an initial inflammatory response, followed by a gradual reduction in macrophages.

A limitation of this trial is that no histological or immunohistochemical analysis was performed during the observation period. However, the absence of clinical inflammation may suggest good soft-tissue integration and minimal pro-inflammatory effects. Additionally, while previous studies have reported slight swelling in similar barriers, no dimensional changes were observed in the disks during the study.

Polymers with β-tricalcium phosphate (TCP) have demonstrated a well-established synergy with oral tissues and a significant ability to induce osteoconductivity. However, little is known about their properties after the printing process and whether they maintain their biological role. Recent in vitro studies [16,29] have shown that co-polymers combined with osteoconductive ceramics can still induce osteoblast differentiation from multipotent mesenchymal cells after printing. Nevertheless, this study represents the first clinical evaluation of such materials and might serve as preliminary evidence for their use in major bone augmentation procedures, combining affordability, availability, and high precision through customized 3D printing.

## 5. Conclusions

The present study introduced the use of composite co-polymers in clinical practice for socket closure after extractions. Two different bio-copolymers, poli-D-lactic acid with 10% hydroxyapatite and poli-ε caprolactone with 20% β-tricalcium phosphate, were employed.

In conclusion, the study highlights the importance of preserving socket volume by employing sealing systems, and cost-effective composite co-polymers demonstrated promising outcomes in terms of soft-tissue closure and absence of complications. These findings contribute to the potential application of these materials in clinical practice for socket preservation after tooth extractions. Further investigations should explore effects on the total volume of the post-extractive site and conduct histological and immune-histochemical analyses to gain a comprehensive understanding of host tissue responses.

## Figures and Tables

**Table 1:** Distribution of the extracted teeth in the 3 groups and exposed area at the beginning and at the end of the observation.

	Test 1	Test 2	Control
Males	7	7	6
Females	6	6	7
Front teeth (inc, canines)	3	3	3
Posterior teeth (premolars-molars)	10	10	10
Initial Exposed Area (mm^2^) T0	46.5 ± 8.25	47.1 ± 8.67	45.6 ± 7.25
Final Exposed Area (mm^2^) T4 (6 weeks)	0.6 ± 0.84	0.6 ± 0.7	1.2 ± 0.9
Difference in exposed area (mm^2^) (T0–T4)	45.9 ± 9.09	46.5 ± 9.37	44.4 ± 8.15

## Data Availability

The datasets used and analysed during the current study are available from the corresponding author on reasonable request.

## References

[1] De Angelis N., Benedicenti S., Zekiy A., Amaroli A. (2022). Current Trends in Bone Augmentation Techniques and Dental Implantology: An Editorial Overview. J. Clin. Med..

[2] Sanz-Sánchez I., Sanz-Martín I., Ortiz-Vigón A., Molina A., Sanz M. (2022). Complications in Bone-grafting Procedures: Classification and Management. Periodontology 2000.

[3] Hardwick R., Hayes B.K., Flynn C. (1995). Devices for Dentoalveolar Regeneration: An Up-to-Date Literature Review. J. Periodontol..

[4] Apaza-Bedoya K., Magrin G.L., Romandini M., Blanco-Carrión J., Benfatti C.A.M. (2023). Efficacy of Alveolar Ridge Preservation with Xenografts and Resorbable Socket Sealing Materials in the Esthetic Region: A Systematic Review with Meta-Analyses. Clin. Implant Dent. Relat. Res..

[5] Juodzbalys G., Stumbras A., Goyushov S., Duruel O., Tözüm T.F. (2019). Morphological Classification of Extraction Sockets and Clinical Decision Tree for Socket Preservation/Augmentation after Tooth Extraction: A Systematic Review. J. Oral Maxillofac. Res..

[6] Majzoub J., Ravida A., Starch-Jensen T., Tattan M., Suárez-López Del Amo F. (2019). The Influence of Different Grafting Materials on Alveolar Ridge Preservation: A Systematic Review. J. Oral Maxillofac. Res..

[7] Canellas J.V.D.S., Medeiros P.J.D., Figueredo C.M.d.S., Fischer R.G., Ritto F.G. (2019). Which Is the Best Choice after Tooth Extraction, Immediate Implant Placement or Delayed Placement with Alveolar Ridge Preservation? A Systematic Review and Meta-Analysis. J. Craniomaxillofac. Surg..

[8] Di Stefano D.A., Orlando F., Ottobelli M., Fiori D., Garagiola U. (2022). A comparison between anorganic bone and collagen-preserving bone xenografts for alveolar ridge preservation: Systematic review and future perspectives. Maxillofac. Plast. Reconstr. Surg..

[9] Flores Fraile J., López-Valverde N., García de Castro Andews A., Santos Marino J.A., Ramírez J.M., Gómez de Diego R., Montero J., López-Valverde A., Blanco Antona L.A. (2020). Safety and Efficacy of a New Synthetic Material Based on Monetite, Silica Gel, PS-Wallastonite, and a Hydroxyapatite Calcium Deficient: A Randomized Comparative Clinic Trial. Medicina.

[10] Jawed S.F., Rabadia C.D., Khan M.A., Khan S.J. (2022). Effect of Alloying Elements on the Compressive Mechanical Properties of Biomedical Titanium Alloys: A Systematic Review. ACS Omega.

[11] Hartmann A., Hildebrandt H., Younan Z., Al-Nawas B., Kämmerer P.W. (2022). Long-Term Results in Three-Dimensional, Complex Bone Augmentation Procedures with Customized Titanium Meshes. Clin. Oral Implants Res..

[12] Cunha G., Carvalho P.H.A., Quirino L.C., Torres L.H.S., Filho V.A.P., Gabrielli M.F.R., Gabrielli M.A.C. (2022). Titanium Mesh Exposure after Bone Grafting: Treatment Approaches-A Systematic Review. Craniomaxillofac. Trauma Reconstr..

[13] Mateo-Sidrón Antón M.C., Pérez-González F., Meniz-García C. (2024). Titanium mesh for guided bone regeneration: A systematic review. Br. J. Oral Maxillofac. Surg..

[14] Kim J., McBride S., Tellis B., Alvarez-Urena P., Song Y.-H., Dean D.D., Sylvia V.L., Elgendy H., Ong J., Hollinger J.O. (2012). Rapid-Prototyped PLGA/β-TCP/Hydroxyapatite Nanocomposite Scaffolds in a Rabbit Femoral Defect Model. Biofabrication.

[15] Moher D., Hopewell S., Schulz K.F., Montori V., Gøtzsche P.C., Devereaux P.J., Elbourne D., Egger M., Altman D.G. (2012). CONSORT 2010 Explanation and Elaboration: Updated Guidelines for Reporting Parallel Group Randomised Trials. Int. J. Surg..

[16] De Angelis N., Amaroli A., Sabbieti M.G., Cappelli A., Lagazzo A., Pasquale C., Barberis F., Agas D. (2023). Tackling Inequalities in Oral Health: Bone Augmentation in Dental Surgery through the 3D Printing of Poly(ε-Caprolactone) Combined with 20% Tricalcium Phosphate. Biology.

[17] Natto Z.S., Parashis A.O., Jeong Y.N. (2020). Soft-Tissue Changes after Using Collagen Matrix Seal or Collagen Sponge with Allograft in Ridge Preservation: A Randomized Controlled Volumetric Study. J. Oral Implantol..

[18] Puppi D., Chiellini F. (2020). Biodegradable Polymers for Biomedical Additive Manufacturing. Appl. Mater. Today.

[19] Farah S., Anderson D.G., Langer R. (2016). Physical and Mechanical Properties of PLA, and Their Functions in Widespread Applications—A Comprehensive Review. Adv. Drug Deliv. Rev..

[20] Song X., Guan W., Qin H., Han X., Wu L., Ye Y. (2022). Properties of Poly(Lactic Acid)/Walnut Shell/Hydroxyapatite Composites Prepared with Fused Deposition Modeling. Sci. Rep..

[21] Ohura K., Bohner M., Hardouin P., Lemaître J., Pasquier G., Flautre B. (1996). Resorption of, and Bone Formation from, New Beta-Tricalcium Phosphate-Monocalcium Phosphate Cements: An in Vivo Study. J. Biomed. Mater. Res..

[22] Penel G., Leroy N., Van Landuyt P., Flautre B., Hardouin P., Lemaître J., Leroy G. (1999). Raman Microspectrometry Studies of Brushite Cement: In Vivo Evolution in a Sheep Model. Bone.

[23] Tamimi F., Torres J., Bassett D., Barralet J., Cabarcos E.L. (2010). Resorption of Monetite Granules in Alveolar Bone Defects in Human Patients. Biomaterials.

[24] Borkar T., Goenka V., Jaiswal A.K. (2021). Application of Poly-ε-Caprolactone in Extrusion-Based Bioprinting. Bioprinting.

[25] Azimi B., Nourpanah P., Rabiee M., Arbab S. (2014). Poly (∊-Caprolactone) Fiber: An Overview. J. Eng. Fiber Fabr..

[26] Koch F., Thaden O., Conrad S., Tröndle K., Finkenzeller G., Zengerle R., Kartmann S., Zimmermann S., Koltay P. (2022). Mechanical Properties of Polycaprolactone (PCL) Scaffolds for Hybrid 3D-Bioprinting with Alginate-Gelatin Hydrogel. J. Mech. Behav. Biomed. Mater..

[27] De Angelis N., Kassim Z.H., Mohd Yusof E., Yumang C., Menini M. (2023). Bone Augmentation Techniques with Customized Titanium Meshes: A Systematic Review of Randomized Clinical Trials. Open Dent. J..

[28] Yu X., Teng F., Zhao A., Wu Y., Yu D. (2022). Effects Of Post-Extraction Alveolar Ridge Preservation Versus Immediate Implant Placement: A Systematic Review and Meta-Analysis. J. Evid. Based Dent. Pract..

[29] García-Lamas L., Sánchez-Salcedo S., Jiménez-Díaz V., Bravo-Giménez B., Cabañas M.V., Peña J., Román J., Jiménez-Holguín J., Abella M., Desco M. (2023). Desing and comparison of bone substitutes. Study of in vivo behavior in a rabbit model. Rev. Esp. Cir. Ortop. Traumatol..

